# TFBMiner: A User-Friendly Command Line Tool for the Rapid Mining of Transcription Factor-Based Biosensors

**DOI:** 10.1021/acssynbio.2c00679

**Published:** 2023-04-13

**Authors:** Erik K. R. Hanko, Tariq A. Joosab Noor Mahomed, Ruth A. Stoney, Rainer Breitling

**Affiliations:** Manchester Institute of Biotechnology, Faculty of Science and Engineering, University of Manchester, 131 Princess Street, Manchester M1 7DN, U.K.

**Keywords:** biosensor, genome mining, bioinformatics, transcriptional regulator, bioengineering, mandelate

## Abstract

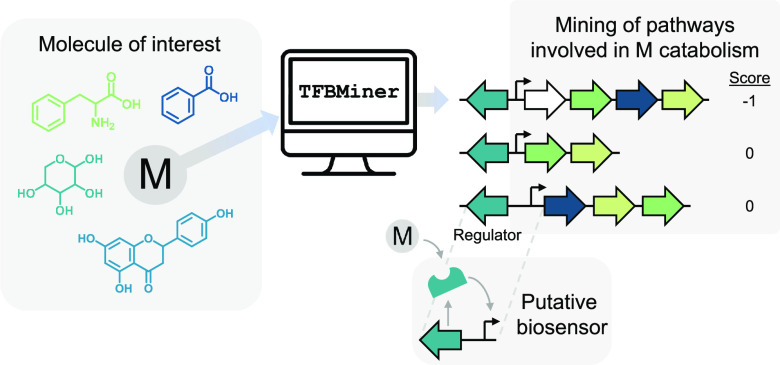

Transcription factors responsive to small molecules are essential elements in synthetic biology designs. They are often used as genetically encoded biosensors with applications ranging from the detection of environmental contaminants and biomarkers to microbial strain engineering. Despite our efforts to expand the space of compounds that can be detected using biosensors, the identification and characterization of transcription factors and their corresponding inducer molecules remain labor- and time-intensive tasks. Here, we introduce TFBMiner, a new data mining and analysis pipeline that enables the automated and rapid identification of putative metabolite-responsive transcription factor-based biosensors (TFBs). This user-friendly command line tool harnesses a heuristic rule-based model of gene organization to identify both gene clusters involved in the catabolism of user-defined molecules and their associated transcriptional regulators. Ultimately, biosensors are scored based on how well they fit the model, providing wet-lab scientists with a ranked list of candidates that can be experimentally tested. We validated the pipeline using a set of molecules for which TFBs have been reported previously, including sensors responding to sugars, amino acids, and aromatic compounds, among others. We further demonstrated the utility of TFBMiner by identifying a biosensor for S-mandelic acid, an aromatic compound for which a responsive transcription factor had not been found previously. Using a combinatorial library of mandelate-producing microbial strains, the newly identified biosensor was able to distinguish between low- and high-producing strain candidates. This work will aid in the unraveling of metabolite-responsive microbial gene regulatory networks and expand the synthetic biology toolbox to allow for the construction of more sophisticated self-regulating biosynthetic pathways.

## Introduction

Genetically encoded biosensors based on transcriptional regulator proteins are one of the most widely used tools in synthetic biology. Their ability to regulate gene expression in response to small molecules has been harnessed in numerous biotechnology applications, including real-time monitoring of metabolite production,^1,2^ optimization of biosynthetic pathways by dynamic regulation,^3,4^ high-throughput enzyme and strain screening,^5,6^ diagnostics,^7^ and environmental monitoring.^8^ However, despite their potential to assist in overcoming a range of engineering and analytical challenges, the number of known biosensors is relatively small in comparison to the number of chemical target molecules.

To expand the universe of compounds that can be detected using transcription factor-based biosensors (TFBs), a range of different approaches has been developed in recent years. Screening for ligand–sensor cross-reactivity using already existing libraries of TFBs is a simple yet effective strategy for identifying sensors for compounds of interest.^9^ The same concept of regulator unspecificity is adopted by Sensbio.^10^ Based on structural similarities, this online tool searches for potential transcription factors for a particular input molecule in a database of recognized regulator–ligand pairs.^10^ Once an initial interaction between a small molecule and a transcription factor has been established, regulator engineering can be employed to improve ligand specificity. This method has been successful in creating biosensors for compounds that are structurally similar to the natural inducer molecules of known TFBs.^11−13^ Sensipath is another online tool that metabolically links compounds for which biosensors have not yet been discovered to molecules for which TFBs currently exist.^14^ It suggests a set of enzymatic reactions which must ultimately be implemented as an integrated component to the biosensor in order to convert a nondetectable molecule into a detectable one. This approach was recently developed further by harnessing hydrogen peroxide as a universal signaling molecule. Because this ubiquitous metabolite is generated by a number of enzymatic reactions, PeroxiHUB eliminates the requirement for target molecule-specific transcriptional regulators.^15^ Although these strategies have considerably contributed to expanding the space of compounds that can be detected using TFBs, their implementation often remains complicated, time-consuming, and labor-intensive.

Remarkably, the vast majority of experimentally validated TFBs derive from a limited number of well-studied species. This may not come as a surprise given that transcriptional regulation is a complex biological process that still requires a basic set of genetic tools and time to unravel. Because of this, the great number of genomes that have become available due to advances in genomics may provide a largely underutilized source of biological activities, including transcriptional regulation in response to a variety of environmental stimuli. This may also indicate that the inducer molecules for a substantial number of transcription factors are yet to be identified. Genome mining is still considered one of the best approaches for discovering novel TFBs because a ligand cannot yet be inferred from a regulator’s protein structure alone.^16^ For example, new metabolite-responsive TFBs have recently been identified by manual screening of the genome of *Cupriavidus necator*.^9^ Based solely on the genomic location of transcriptional regulator genes in relation to putative ligand-catabolizing operons, inducer molecules were assigned to transcription factors. Although a total of 15 biosensors were experimentally validated,^9^ the manual search for regulator–operon pairs remains time-consuming, and the ligands for which sensors can be found are limited to the ligand-metabolizing pathways present in a particular genome.

In this work, we address these challenges by developing a tool for the automated mining of metabolite-responsive TFBs across thousands of genomes simultaneously. This easy-to-use command line tool identifies potential biosensors for user-defined target metabolites by leveraging a heuristic rule-based model of gene organization. The tool is validated using a set of compounds for which TFBs have been previously reported. As a proof of concept, we employ the tool to mine putative biosensors for the aromatic compound mandelic acid. We demonstrate that an *Escherichia coli* whole-cell biosensor carrying the *Paraburkholderia hospita* MdlR/P_*mdlC*_-inducible system responds to S-mandelate in a dose-dependent manner. The user-friendly tool presented here will enable the rapid mining of novel metabolite-responsive TFBs, thereby expanding the universe of detectable molecules.

## Results

### Development of a Pipeline for Mining Transcription Factor-Based Biosensors

Two biological elements need to be identified when developing transcription factor-based biosensors (TFBs): (i) the transcriptional regulator protein, which binds the desired molecule, and (ii) the operator sequence, with which the regulator interacts. Recently, we harnessed a heuristic rule-based model of gene organization for the genome-wide identification of biosensors in *Cupriavidus necator*.^9^ In that study, we manually screened the genome of *C. necator* for catabolic operons that are transcribed in the direct opposite orientation of transcriptional regulators, which, solely based on their location, were predicted to have a role in controlling the expression of these operons. By comparing the metabolic substrates and products of the enzymes encoded within an operon, as well as considering previously reported regulons of the same genetic arrangement, inducer molecules were inferred. Following this approach, we identified 16 putative biosensors, 15 of which were experimentally verified.^9^ To automate this workflow and expand the search to a larger set of genomes, using user-defined compounds as input, TFBMiner was developed.

**Figure 1:**
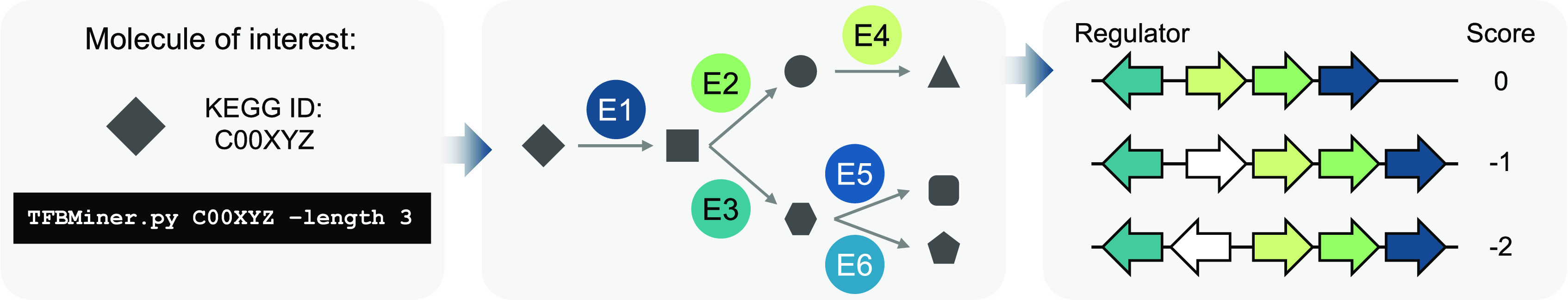
Schematic of the TFBMiner workflow. When given the KEGG compound ID of the molecule of interest and the number of enzymatic steps that are to be encoded by the catabolic operon (e.g., a chain length of 3), TFBMiner retrieves all enzymatic chains that would result in the sequential processing of the target molecule. A separate output file is generated for each chain for which TFBMiner is able to identify the individual catalytic steps to be encoded within a single genome and cluster the genes encoding these functions based on their genomic location. A score is calculated based on the number of genes that are located between the putative target compound-metabolizing operon and the nearest transcriptional regulator encoded in the opposite orientation. E: enzyme.

As its name implies, TFBMiner facilitates the mining of both operons and regulators that are involved in the catabolism of user-specified molecules and ranking them based on how well they fit the above-mentioned heuristic model of gene organization. An overview of the TFBMiner workflow is illustrated in Figure 1. The command line tool requires two inputs from the user: the Kyoto Encyclopedia of Genes and Genomes (KEGG) compound ID of the target molecule, and the number of catalytic steps that are to be encoded by the catabolic operon. When given both inputs, TFBMiner records all enzymatic reactions that are involved in catabolizing the specified compound, including relevant products. This step is repeated using each of the resulting product molecules as a new input until the maximum number of catalytic steps, as defined by the user, is reached. As a result, for each target molecule, TFBMiner may generate multiple chains of enzymatic reactions, each chain presenting a unique sequence of reactions. Once a set of enzymatic chains has been identified, a comprehensive library of bacterial genomes is screened for the individual catalytic steps. In case a genome encodes all relevant enzymes belonging to an enzymatic chain, TFBMiner determines whether they are likely to belong to an operon by mapping their corresponding genes. Consequently, for each enzymatic chain for which an operon can be identified, TFBMiner generates a separate output file. Each output file comprises KEGG organism identifiers, locus tags of both the genes that encode the individual enzymatic reactions and their putative transcriptional regulators, as well as regulator annotations. Subsequently, a score for each regulator–operon pair is provided, which is calculated based on proximity of the operon to the regulator gene. Operons that are transcribed in the direct opposite orientation of transcriptional regulators are assigned the highest score, 0. For each gene located between the putative operon and the nearest regulator in opposite orientation, points are subtracted. Scores can range from 0 to −500, with −500 being the predefined limit. Regulators with a high score are more likely to be involved in activation of operon expression in response to the target molecule, whereas regulators with a low score are predicted to have a lower probability of being functionally related to the operon. Therefore, the score serves as guidance, helping to limit the number of biosensors that the user has to test experimentally.

In many cases, selecting an enzymatic chain length of 2 or 3, catalyzing 2 or 3 steps, respectively, is sufficient for the pipeline to reliably return operons that are involved in the sequential processing of a specified target compound. The longer the enzymatic chain selected by the user, the more catalytic functions an operon must encode to be identified by TFBMiner. Although selecting a longer enzymatic chain may increase the likelihood of the returned sets of genes to be co-expressed in response to a user-defined molecule, many compounds may require fewer catalytic steps to be converted into primary metabolites. Alternatively, the downstream part of a degradation pathway may be shared between compounds and thus be encoded by a separate operon. Consequently, selecting an adequate number of catalytic steps that are to be encoded by the catabolic operon may require some trial and error. By default, TFBMiner will return putative biosensors for the specified enzymatic chain length in addition to any shorter chains with a length of at least 2 reactions to reduce the need for ad hoc optimization.

### Validation of the Tool Using Molecules with Known TFBs

To validate the ability of TFBMiner to accurately identify target compound-catabolizing pathways and rank them based on their genetic proximity to transcriptional regulator genes, 31 compounds for which biosensors have been reported previously were tested with a chain length set to 3. These compounds include a diverse range of sugars, amino acids, dicarboxylic acids, and aromatic carboxylic acids, among others (Table 1).^17^ The number of identified enzymatic chains and putative biosensors was recorded for each compound. All biosensors that received a score of 0 are listed in Supplementary File S2.

**Table 1:** Compounds Tested To Validate TFBMiner^a^

ligand	KEGG compound ID	source	regulator	identified chains	identified biosensors	reference
original biosensor was identified						
β-l-arabinose	C02479	*E. coli*	AraC	3	2095	(18)
l-arginine	C00062	*B. licheniformis*	RocR	994	10,828	(19)
benzoate	C00180	*A. baylyi*	BenM	70	2488	(20)
benzoate	C00180	*C. necator*	BenM	70	2488	(9)
glutarate	C00489	*P. putida*	CsiR	33	435	(21)
l-kynurenine	C00328	*C. necator*	KynR	558	2688	(9)
l-phenylalanine	C00079	*C. necator*	PhhR	1175	1955	(9)
l-phenylalanine	C00079	*P. aeruginosa*	PhhR	1175	1955	(22)
phenylglyoxylate	C02137	*C. necator*	PhgR	25	367	(9)
l-proline	C00148	*R. leguminosarum*	PutR	159	6480	(23)
salicylate	C00805	*C. necator*	NahR	84	380	(9)
tartrate	C00898	*C. necator*	TtdR	273	8523	(9)
l-tyrosine	C00082	*C. necator*	HpdA	1140	11,622	(9)
vanillate	C06672	*C. crescentus*	VanR	305	505	(24)
xanthine	C00385	*C. necator*	XdhR	12	3131	(9)
d-xylose	C00181	*P. megaterium*	XylR	19	8307	(25)
original biosensor was not identified: resemblance of the ligand’s structure to that of the original inducer molecule						
ε-caprolactam	C06593	*Acinetobacter* sp. NCIMB 9871	ChnR	0	0	(26)
isoprene	C16521	*R. pickettii*	TbuT	0	0	(27)
original biosensor was not identified: operon regulation is unrelated to ligand metabolism						
N-(3-oxohexanoyl)-l-homoserine lactone	C21198	*V. fischeri*	LuxR	0	0	(28)
naringenin	C00509	*P. putida*	TtgR	114	0	(29)
original biosensor was not identified: the KEGG database is incomplete (compounds listed as products rather than substrates; gene entries not linked to entries for enzymes or organisms; strains or enzymes not listed)						
3-hydroxypropanoate	C01013	*C. necator*	HpdR	68	14	(30)
β-alanine	C00099	*C. necator*	OapR	213	2904	(9)
cyclohexane-1-carboxylate	C09822	*C. necator*	BadR	0	0	(9)
glutarate	C00489	*P. putida*	GcdR	33	435	(21)
l-isoleucine	C00407	*P. putida*	BkdR	110	955	(31)
naringenin	C00509	*H. seropedicae*	FdeR	114	0	(32)
progesterone	C00410	*P. simplex*	SRTF1	52	0	(33)
sulfoacetate	C14179	*C. necator*	SauR	0	0	(9)
original biosensor was not identified: gene organization does not match the required model						
acetoin	C00466	*C. necator*	AcoR	28	0	(9)
acrylate	C00511	*R. sphaeroides*	AcuR	2	150	(34)
choline	C00114	*E. coli*	BetI	363	2894	(35)
erythromycin A	C01912	*E. coli*	MphR	0	0	(36)
glucarate	C00818	*E. coli*	CdaR	22	1159	(37)
oleate	C00712	*E. coli*	FadR	32	0	(38)
l-phenylalanine	C00079	*E. coli*	TyrR	1175	1955	(39)
putrescine	C00134	*E. coli*	PuuR	362	1319	(40)
l-tyrosine	C00082	*E. coli*	TyrR	1140	11,622	(39)

a The most frequent issues that result in the tool’s inability to find the reported biosensors are outlined.

**Figure 2:**
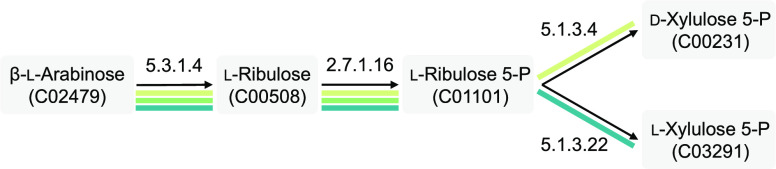
Possible enzymatic chains involved in the sequential catabolism of β-l-arabinose. TFBMiner predicted three enzymatic chains in total: one with a chain length of two reactions and two with a chain length of three reactions. Enzyme commission numbers and KEGG compound IDs are indicated.

For example, β-l-arabinose is the substrate of the well-known arabinose-utilization operon in *Escherichia coli*, the expression of which is controlled by AraC.^18^ When given the KEGG compound ID of β-l-arabinose and a chain length of 3 as inputs, TFBMiner returns a total of three enzymatic chains (Table 1, Figure 2). All three chains comprise l-arabinose isomerase (EC 5.3.1.4) and ribulokinase (EC 2.7.1.16), converting β-l-arabinose into l-ribulose 5-phosphate. The first and second chains additionally contain l-ribulose-5-phosphate 4-epimerase (EC 5.1.3.4) or l-ribulose-5-phosphate 3-epimerase (EC 5.1.3.22), converting l-ribulose 5-phosphate into the d- or l-isomer of xylulose 5-phosphate, respectively. The third chain comprises only the first two enzymes. For each chain, TFBMiner is able to generate a separate output file, suggesting that putative operons and their associated transcriptional regulator genes were identified. For example, the chain encoding 5-phosphate 4-epimerase yields 870 potential biosensors with a score of greater than −500, 265 of which have an ideal score of 0 (Supplementary File S2). Among these 265 identified putative biosensors, 167 have associated regulator genes that are annotated to encode either a transcriptional regulator AraC or an arabinose operon regulatory protein, indicating that TFBMiner correctly returned β-l-arabinose-catabolizing pathways and their associated transcriptional regulator proteins. One of them is the *E. coli* K-12 MG1655 AraC/P_*araBAD*_-biosensor. It should be highlighted that other β-l-arabinose sensors with a score of 0 utilize regulator proteins belonging to alternative families of transcription factors, including LacI- and GntR-type regulators, both of which have been reported to be involved in mediating arabinose catabolism.^41,42^ This demonstrates that TFBMiner can also be utilized to identify biosensors composed of alternative regulator proteins. As they may differ in their induction kinetics and dynamics, these alternative biosensors could overcome drawbacks, such as a limited detection range or a narrow dose–response relationship, reported for previously established sensors responding to the same molecule.

The same accurate predictions were achieved for a further 13 out of the 31 investigated compounds (Table 1). One or more of the following reasons were found to be the cause of a previously reported biosensor not being returned: (i) the ligand is not the original inducer molecule but is able to interact with the transcription factor due to regulator promiscuity, (ii) the operon is involved in biological processes other than metabolism, such as ligand transport, (iii) the KEGG database is incomplete, and (iv) the genetic organization of the transcriptional regulator and operon does not fit the heuristic model used by TFBMiner. In some of these cases, TFBMiner was able to suggest alternative biosensors that had been experimentally validated. For instance, TyrR, an *E. coli* transcriptional regulator that controls the expression of multiple transcription units in response to l-phenylalanine and l-tyrosine, was not returned since it is not encoded in the opposite orientation of any of these units.^39^ However, TFBMiner correctly identified the l-phenylalanine and l-tyrosine biosensors from *C. necator*. Moreover, TFBMiner was able to predict potential biosensors for a variety of molecules, including acrylate, β-alanine, choline, glucarate, glutarate, l-isoleucine, and putrescine, which have not yet undergone experimental confirmation (Supplementary File S2).

### Application of TFBMiner To Identify an S-Mandelate-Responsive Biosensor

To demonstrate that TFBMiner can be harnessed to predict biosensors for compounds for which sensors had not been identified previously, the tool was tested for the aromatic compound mandelic acid. Both enantiomers of this molecule are important chiral building blocks used for the synthesis of a wide range of pharmaceuticals, including anti-cancer, anti-obesity, and anti-inflammatory drugs;^43^ a biosensor could offer important benefits for the high-throughput engineering of mandelic acid-producing microbial cell factories. Furthermore, mandelic acid is employed as a urinary biomarker, indicating exposure to the volatile organic compounds ethylbenzene and styrene.^44^ As a result, the development of inexpensive and user-friendly methods for the detection and quantification of mandelic acid in biological samples is an active field of research.^45^

**Figure 3:**
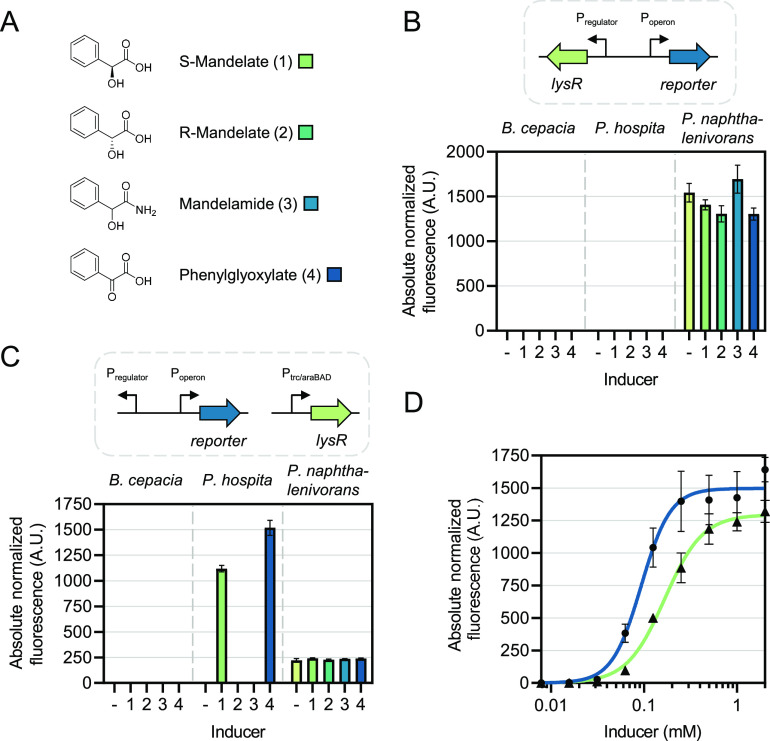
Identification and characterization of a biosensor responding to S-mandelate. (A) Chemical structures of both enantiomers of mandelate, as well as metabolically related compounds mandelamide and phenylglyoxylate. (B) and (C) Absolute normalized fluorescence of the *E. coli* whole-cell biosensors in response to the compounds S-mandelate (1), R-mandelate (2), mandelamide (3), and phenylglyoxylate (4). Biosensors consist of two parts. The first part comprises the LysR-type transcriptional regulator gene under control of (B) its native promoter or (C) a *trc* or *araBAD* promoter. Part two comprises the *rfp* reporter gene linked to the promoter of its corresponding operon, activation of which is putatively controlled by the regulator. Biosensors are derived from *Burkholderia cepacia*, *Paraburkholderia hospita*, and *Polaromonas naphthalenivorans*. Single time-point fluorescence measurements were taken 6 h after supplementation with an inducer to a final concentration of 0.5 mM. (D) Dose–response curves of *E. coli* carrying both *P. hospita* MdlR under control of P_*trc*_ and *rfp* under control of *P. hospita* P_*mdlC*_ in response to different concentrations of S-mandelate and phenylglyoxylate. Single time-point fluorescence measurements were taken 6 h after supplementation with inducer. The dose–responses were fit using a Hill function. (−) uninduced sample. Error bars represent standard deviations of three biological replicates.

Despite its well-characterized degradation pathway in both prokaryotes and eukaryotes, little is known about the transcriptional regulation of mandelic acid catabolism,^46^ posing a challenge to the development of mandelic acid-responsive genetically encoded biosensors. Here, the TFBMiner workflow was applied to both enantiomers of this molecule. Although 14 enzymatic chains with a length of ≤3 reactions were returned for R-mandelate, no potential biosensor was identified (Table S1 in Supplementary File S1). For S-mandelate, TFBMiner was able to retrieve 325 enzymatic chains and generate output files for 48 of them with a length of 3 enzymatic steps. For ten of the 48 chains, biosensors with an optimal score of 0 were obtained (Table S2 in Supplementary File S1). Ultimately, nine of them were manually eliminated as their second reactions solely regenerate the cofactor that is required by the first enzyme, resulting in a loop of enzymatic reactions for chains that are longer than 2 steps. For the remaining chain, TFBMiner identified 13 biosensors with a score of 0 (Table S3 in Supplementary File S1). This chain comprises (*S*)-mandelate dehydrogenase, which oxidizes S-mandelate to phenylglyoxylate; phenylglyoxylate decarboxylase, converting phenylglyoxylate into benzaldehyde; and benzaldehyde dehydrogenase, oxidizing benzaldehyde to benzoate (Figure S1 in Supplementary File S1). Interestingly, all 13 regulators are annotated as belonging to the family of LysR-type transcriptional regulators, which are known to be often encoded in the opposite orientation of the cluster of genes that they regulate.^47^ A regulator protein sequence alignment was performed to minimize the number of screening candidates by eliminating proteins that share a high sequence similarity to other proteins in the candidate set (Figure S2 in Supplementary File S1). This reduced the number of biosensors that needed to be experimentally tested for a change in gene expression in response to S-mandelate. The final set of candidates included 3 nonredundant regulators from *Burkholderia cepacia*, *Paraburkholderia hospita*, and *Polaromonas naphthalenivorans*. Regulator genes, optimized for *E. coli* codon usage, and their corresponding intergenic regions, harboring the putative S-mandelate-inducible promoters, were synthesized and cloned into a reporter vector. The original genetic arrangement was maintained by inserting the regulator gene in the opposite orientation of the *rfp* reporter gene (Figure S3 in Supplementary File S1). *E. coli* strains carrying the three reporter vectors were grown in minimal medium, and the RFP fluorescence output was monitored in the absence and presence of inducers. In addition to S-mandelate, the metabolically related compounds R-mandelate, mandelamide, and phenylglyoxylate were tested (Figure 3A). Whereas the *P. naphthalenivorans* biosensor exhibited a basal level of gene expression, which remained unchanged in the presence of any of the four inducers, the other two sensors did not show any fluorescence (Figure 3B). To determine whether the lack of inducible gene expression is caused by the absence of the regulator protein, we expressed the regulator genes separately using promoters that are well-characterized in *E. coli*. The regulators from *P. hospita* and *B. cepacia* were expressed using an IPTG-controllable *trc* promoter. *P. naphthalenivoransPnap_*1024 was expressed using an arabinose-controllable promoter, which has been shown to be regulated more tightly than P_*trc.*_^48^*E. coli* strains carrying both the regulator genes under control of either IPTG- or arabinose-inducible promoters and their corresponding mandelate-inducible promoters linked to *rfp* were tested for fluorescence output in the absence and presence of inducers. This time, the *E. coli* whole-cell biosensor carrying *P. hospita* C2L64_43720/P_*C*2*L*64*_*43715_ showed an activation of reporter gene expression when supplemented with either S-mandelate or phenylglyoxylate, whereas addition of R-mandelate or mandelamide did not result in RFP fluorescence (Figure 3C). The whole-cell biosensors harboring *B. cepacia* APZ15_29770/P_*APZ*15*_*29775_ and *P. naphthalenivorans* Pnap_1024/P_*Pnap_*1023_ demonstrated no induction of *rfp* expression.

To determine the operational range of *P. hospita* C2L64_43720/P_*C*2*L*64*_*43715_, hereafter termed *P. hospita* MdlR/P_*mdlC*_, in response to S-mandelate and phenylglyoxylate, we supplemented *E. coli* cultures carrying both the sensing and reporter modules with varying inducer concentrations and quantified the reporter output (Figure 3D). Subsequently, data points were fit using a Hill function to obtain inducer-specific parameters characterizing the individual dose–response curves (Table 2). As indicated by the Hill coefficients, the operational range of *P. hospita* MdlR/P_*mdlC*_ in response to S-mandelate is wider than that for phenylglyoxylate. Here, the operational range was defined as the inducer titer range corresponding to 5–95% of the maximum level of the reporter output. For S-mandelate, this range spans from 42 to 665 μM, whereas for phenylglyoxylate, it spans from 32 to 270 μM.

**Table 2:** Parameters of the *P. hospita* MdlR/P_*mdlC*_-Based Whole-Cell Biosensor in Response to S-Mandelate and Phenylglyoxylate

			operational range	
inducer	Hill coefficient, *h*	*K*_m_^a^ (μM)	*I* (5% *b*_max_) (μM)	*I* (95% *b*_max_) (μM)
S-mandelate	2.13	167	42	665
phenylglyoxylate	2.76	93	32	270

a Inducer concentration at which the half-maximal reporter output (*b*_max_) is achieved.

### Detection of S-Mandelate in Biological Samples Using *P. hospita* MdlR/P_*mdlC*_

The range of applications for biosensors like the one presented here is extremely diverse. For example, it can be utilized to detect and quantify its corresponding ligand when supplied exogenously like in the case of testing clinical samples. Moreover, it can be deployed within an organism that produces the ligand in order to monitor its concentration in real time. The latter strategy is particularly useful when the biosensor is used to screen for strains with improved capacities to synthesize the target using techniques like fluorescence-activated cell sorting. Because mandelic acid is employed as a urinary biomarker, we sought to evaluate how well the *P. hospita* MdlR/P_*mdlC*_-based biosensor performs when exposed to biologically generated extracts containing S-mandelate. To test this, we harnessed the whole-cell biosensor to distinguish between five strains of *E. coli* that have been reported to biosynthesize S-mandelate at varying levels.^49^

**Figure 4:**
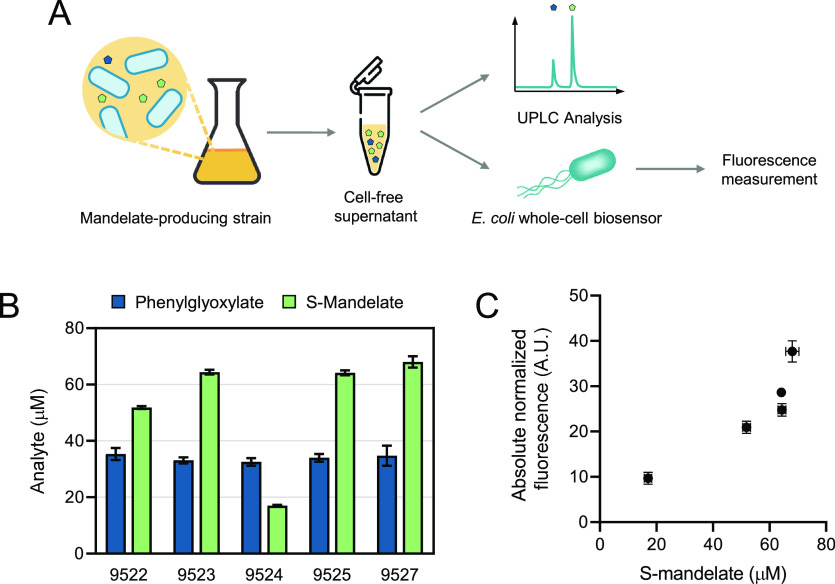
Application of the *E. coli* whole-cell biosensor for detection and quantification of S-mandelate in biological samples. (A) Schematic of the workflow for the detection and quantification of S-mandelate in culture supernatants of mandelate-producing microbial strains using UPLC or the *E. coli* whole-cell biosensor. (B) Final concentrations of S-mandelate and phenylglyoxylate after being added to the cultures containing the *E. coli* whole-cell biosensor. Identifiers of mandelate-producing strains are given. (C) Cell-free supernatants of five *E. coli* strains, producing different levels of S-mandelate, were added to the whole-cell biosensor cultures at a ratio of 1:9. Absolute normalized fluorescence levels were determined 12 h after supplementation with the cell-free mandelate-containing supernatants. Error bars represent standard deviations of three biological replicates.

The supernatants of the mandelate-producing strains were used to (i) quantify the levels of both mandelate enantiomers and phenylglyoxylate by conventional analytical techniques and (ii) quantify the fluorescence output after addition to the *E. coli*-based whole-cell biosensor (Figure 4A). Based on the S-mandelate concentrations determined by ultra-performance liquid chromatography (UPLC, Figure 4B), S-mandelate containing supernatants were added to the whole-cell biosensor cultures at a ratio of 1:9 to be within the linear range of detection. Despite the five *E. coli* strains producing a uniform level of phenylglyoxylate (Figure 4B), which can result in a baseline fluorescent protein reporter output in all samples, a clear correlation between S-mandelate titers quantified by UPLC and biosensor response can be observed (Figure 4C). These results indicate that the *E. coli*-based whole-cell biosensor can be harnessed to detect and quantify S-mandelate in biological samples, which paves the way for future applications in clinical diagnostics and biotechnology.

## Discussion

Transcription factor-based biosensors that control gene expression in response to small molecules have proven to be powerful genetic tools across all fields of biotechnology.^50,51^ However, the number of compounds with suitable biosensors is small compared to the multitude of metabolites found in living organisms. As a result, the lack of TFBs for user-specific molecules limits their widespread application in synthetic biology. In this work, we presented a user-friendly bioinformatics tool that allows for the rapid mining of TFBs. TFBMiner enables the automated screening of thousands of genomes for biosensors that respond to user-defined molecules. This is the first attempt to mine TFBs from the largely untapped source of metabolite-induced transcriptional regulation throughout the prokaryotic kingdom.

The current version of TFBMiner has been optimized for commonly found gene arrangements in which the expression of metabolite degradation pathways is controlled by divergently encoded transcriptional regulators and the primary inducer molecule is the most upstream metabolite, which is broken down by the catabolic operon. Even though this is a very common arrangement for gene clusters whose expression is controlled by small molecules, like in the case of several catabolic operons regulated by LysR-type transcriptional regulators,^47^ not all biosensors follow this pattern. Some situations where TFBMiner is less suitable are those in which transcriptional regulators control their own expression as a component of an operon that encodes a metabolite degradation pathway. Moreover, expressions may be initiated in response to molecules that are metabolically distant or unrelated, intermediate compounds, or the final product of the catabolic cascade. For instance, the transcriptional regulator of the *Rhodobacter sphaeroides* dimethylsulfoniopropionate degradation pathway is encoded by the first gene of the three-gene operon, the expression of which is initiated in response to the final product, acrylate.^34^ Another example is the benzoate-responsive transcription factor BenR from *Pseudomonas putida*. The gene that encodes BenR is located directly adjacent to the benzoate catabolic operon.^52^ TFBMiner was able to predict the chain of enzymes that convert benzoate to catechol; however, *benR* from *P. putida* was not found since it is encoded on the same strand as the benzoate catabolic operon. Instead, TFBMiner identified the benzoate-responsive regulator BenM from *A. baylyi* and *C. necator*, indicating that even though some sensors might be missed due to their genetic arrangement, TFBMiner can return alternative candidates as it screens a large number of genomes, some of which may fit the currently employed model of gene organization. While screening a wide range of genomes increases the likelihood of discovering a biosensor, it also increases the possibility of false positives being returned. Catabolic operons may be unregulated in some circumstances, such as xenobiotic-degrading pathways,^53^ and as a result, biosensors may not be present. Filters could be implemented in future to only return transcription factors that are actively involved in target compound-mediated control of gene expression once the DNA binding sites of transcriptional regulators can be predicted from their protein structure.^54^

The ability of TFBMiner to propose alternative biosensors for molecules for which biosensors already exist is an additional benefit. It can suggest both homologs of previously established transcription factors, but also more interestingly, regulators from different families. The majority of biotechnology applications require biosensors to operate within a defined ligand concentration range, the tuning of which is frequently achieved by altering the regulator’s binding affinity to its ligand and/or operator sequence.^55^ Leveraging alternative transcription factors to extend the detection range can be a more feasible approach, the exploration of which will be greatly facilitated by TFBMiner.

The comprehensiveness of the dataset that TFBMiner uses to predict potential metabolite-catabolizing pathways determines how well it is able to discover biosensors for user-defined compounds. Because it is one of the most utilized collections of integrated databases, KEGG was selected to obtain the data necessary for the identification of enzymatic chains. It contains an extensive record of known genomes, enzymes, reactions, and compounds, all of which are assigned unique identifiers that enable the data entries to be accurately linked. In some instances, biosensors were missed by the mining pipeline even though they met the requirements to be returned. This was often the case when genes, reactions, or genomes were absent from the dataset. For example, TFBMiner was unable to identify a biosensor for R-mandelate. This was unexpected since, except for the first step, which involves the interconversion of R- and S-mandelate by the action of a mandelate racemase,^56^ both enantiomers of mandelate share the same degradation pathway. Because R-mandelate is only listed as a product of the racemase enzyme on KEGG, TFBMiner was unable to generate an enzymatic chain starting with the conversion of R- into S-mandelate. Although future database updates will most likely solve this problem, as well as result in the discovery of an even wider range of biosensors, the tool’s capacity to mine sensors for user-defined molecules is currently limited to whether a catabolic pathway for the compound exists and whether expression of this pathway is regulated at the transcriptional level. The tool is particularly helpful for small molecules, which prokaryotes typically metabolize to be used as a source of energy or carbon. Natural target-responsive transcriptional regulators may not exist for more complex molecules, including structurally diverse secondary metabolites, or if they do, they may be involved in controlling nonmetabolic processes that provide the organism a selective advantage. In situations like these, a combination of biosensor mining, *in vivo* and *in silico* library screening, followed by rational regulator engineering, could yield designer biosensors covering the desired detection space.

Finally, we demonstrated that TFBMiner can assist in unraveling metabolite-responsive control of gene expression. Here, we showed that *P. hospita* MdlR activates gene expression in response to S-mandelate and phenylglyoxylate, shedding light on the biological signaling process responsible for mandelic acid catabolism in bacteria. Investigating potential regulatory mechanisms involved in the catabolism of other metabolites can be accomplished by employing the same approach. Interestingly, S-mandelate elicited a response mediated by *P. hospita* MdlR, but not the R-enantiomer. This finding is crucial as it allows for the evolution of mandelate-producing enzymes that prefer one enantiomer over the other using the MdlR biosensor.

The extent of genetic tools at our disposal substantially influences our capacity to engineer biological systems. Biosensors are one of the most important genetic tools, whether they are used to detect and report the presence of metabolites or to rewire cellular processes as a component of genetic circuits. The TFBMiner genome mining tool that is presented here will contribute significantly to the development of biosensors by broadening both the universe of molecules that can be sensed and responded to, as well as the concentration range at which they operate. It will provide biological engineers a more comprehensive set of genetic switches for monitoring compounds of interest and reprogramming metabolism, especially when combined with other strategies aimed at expanding the sensor space, such as regulator engineering.

## Methods

### Development of TFBMiner and Platform Accessibility

TFBMiner was developed in Python 3.10.1. Its source code and comprehensive usage instructions are maintained in the GitHub repository located at https://github.com/UoMMIB/TFBMiner. TFBMiner is Open Access under an MIT License.

The first step in the mining of TFBs is the identification of operons that are involved in the catabolism of the target compound. To collect the required information, TFBMiner leverages the representational state transfer protocol to execute specific KEGG database queries.^57^ KEGG was selected because it consists of several sub-databases that contain extensive information about genes, compounds, reactions, and enzymes. Each entry has a unique identifier that can be used to cross-reference information between the different sub-databases.

TFBMiner first retrieves all reactions involving the molecule of interest. Using the KEGG COMPOUND ID as a search query, it extracts all KEGG REACTION IDs from the KEGG COMPOUND database. The REACTION IDs are then used as search queries in the KEGG REACTION database to obtain the COMPOUND IDs of the reaction partners. Subsequently, for each reaction, TFBMiner determines whether the compound of interest is a substrate or a product in the reaction. If it is a substrate, TFBMiner stores the REACTION IDs, extracts the Enzyme Commission (EC) numbers of the involved enzymes, and records their products. This process is repeated, this time treating the products of the previous reactions as substrates. TFBMiner links the enzymes that are putatively involved in the sequential processing of the molecule of interest until a maximum chain length of enzymatic reactions is reached. This chain length can be specified by the user.

These enzymatic chains are subsequently used to identify putative catabolic operons. TFBMiner first conducts a query within the KEGG ENZYME database. Using the EC numbers of the involved enzymes as search queries, it extracts all genes that are listed to encode them, as well as the organisms that harbor these genes. It then filters the data to only keep those organisms that contain all genes belonging to a given enzymatic chain. Subsequently, TFBMiner links the KEGG organism codes to their corresponding GenBank assembly accession codes using an internal database. The 6830 bacterial genomes that are listed in the KEGG database (release 101.0) were previously downloaded from the National Centre for Biotechnology Information (NCBI) Assembly database (URL: https://www.ncbi.nlm.nih.gov/assembly) and stored locally. The genomes are organized as feature tables, comprising the required information for operon identification. Using these feature tables, TFBMiner assesses the proximity and strand orientation of the genes encoding an enzymatic chain. It generates a separate output file for each enzymatic chain for which an operon is predicted.

The final step comprises regulator identification and scoring. For each potential operon that is identified, TFBMiner retrieves the nearest transcriptional regulator that is encoded in the opposite orientation. This is done by searching for terms in the annotation column of the genome feature tables that denote a regulatory function, such as “regulator,” “activator,” or “repressor.” Following that, for each operon–regulator pair, a score is calculated based on the gene index positions of the regulator and the operon’s most upstream gene. A regulator is assigned the highest possible score, 0, if it is located adjacent to an operon. Integer points are deducted from this base score for each gene that is located between the regulator and the operon. For each gene with the same strand orientation as the operon, one point is deducted, while for each gene with the same strand orientation as the regulator, two points are deducted. We decided to deduct fewer points if a flanked gene is on the same strand as the catabolic operon because such genes may simply be members of the operon that the software missed.

### Base Strains and Media

New England Biolabs (NEB) 5α competent *E. coli* was used for cloning, plasmid propagation, and evaluation of reporter gene expression. Strain *E. coli* DH5α Δ*tyrR* Δ*tyrA* was employed for the biosynthesis of mandelate.^49^ All strains used in this study are listed in Table S4 in Supplementary File S1. Cells were routinely propagated in Luria-Bertani (LB) medium.^58^ Fluorescence reporter assays were performed in M9 minimal medium^58^ supplemented with 1 μg/mL thiamine, 0.1% (w/v) casamino acids, and 4 g/L glucose. To produce mandelate, cells were grown in phosphate-buffered Terrific Broth (TBP, Formedium) supplemented with 0.4% (v/v) glycerol. When required, antibiotics were added to the media at the following concentrations: 34 μg/mL chloramphenicol, 50 μg/mL kanamycin, and 100 μg/mL carbenicillin. To prepare solid media, 15 g/L agar was added.

### Cloning and Transformation

Plasmid DNA was extracted using the QIAprep Spin Miniprep Kit (Qiagen). DNA was amplified by PCR in 50 μL reactions using the Q5 High-Fidelity 2X Master Mix from NEB. The Zymoclean Gel DNA Recovery Kit (Zymo Research) was employed to extract gel-purified linearized DNA. NEBuilder HiFii DNA Assembly Master Mix and restriction enzymes were purchased from NEB. All PCR-, HiFi-, and digestion reactions were set up following the manufacturer’s instructions. Chemical competent *E. coli* were prepared and transformed by heat shock.^58^

### Plasmid Construction

Oligonucleotide primers were synthesized by IDT and are listed in Table S5 in Supplementary File S1. Intergenic regions, harboring the putative mandelate-inducible promoters, and transcription factor coding sequences, optimized for *E. coli* codon usage, were synthesized by TWIST Bioscience. The sequences of the synthesized DNA fragments can be found in Table S6 in Supplementary File S1. Plasmids were constructed by HiFi DNA Assembly and a detailed description of how each plasmid was built can be found in the Supplementary Methods in Supplementary File S1. Correct assembly was validated by Sanger sequencing (Eurofins Genomics). All plasmids used and generated in this study are listed in Table S7 in Supplementary File S1.

### Quantification of Fluorescence and Optical Density

To measure the fluorescence output at a single time point, individual colonies of freshly transformed bacterial cells were used to inoculate 1 mL of M9 minimal medium, containing the appropriate antibiotics, in 96-deepwell blocks with breathable seals. After incubation overnight with orbital shaking at 850 rpm, 30 °C, and 75% humidity, seed cultures were normalized to OD_600_ = 1.0 before being diluted 1:50 into 1 mL of fresh M9 minimal medium containing the respective antibiotics. Where appropriate, to initiate the expression of the regulator genes, exponentially growing cells with an OD_600_ of 0.05–0.1 were supplemented with isopropyl β-d-1-thiogalactopyranoside (IPTG) or l-arabinose to final concentrations of 100 μM. At the same time, S- or R-mandelate, mandelamide, or phenylglyoxylate was added to achieve final concentrations ranging from 0.0078 to 2.0 mM. After a further incubation at 850 rpm, 30 °C, and 75% humidity for 6 h, 100 μL of cells was transferred to a 96-well microtiter plate (black, flat, and clear bottom; Greiner Bio-One). RFP fluorescence and optical density were quantified using a CLARIOstar microplate reader (BMG LABTECH). Fluorescence excitation and emission wavelengths were set to 585 and 620 nm, respectively. The gain factor was set manually to 1500. Culture optical density was quantified at 600 nm to normalize RFP fluorescence by optical density. To account for media auto-fluorescence and optical density, RFP fluorescence and OD_600_ values were corrected by subtracting the fluorescence and OD_600_ of the cell-free culture medium prior to normalization. Absolute normalized fluorescence was calculated as previously described.^59^ Mathematical modeling to obtain inducer-specific parameters characterizing the individual dose–response curves was performed as previously reported.^9^

To determine the biosensor output in response to mandelate-containing culture supernatants, seed cultures of the whole-cell biosensor were set up as above. The biosensor comprised *E. coli* 5α carrying SBC015878 and SBC015896. After incubation overnight, seed cultures were normalized to OD_600_ = 0.02 in 5 mL of fresh M9 minimal medium and grown with orbital shaking at 30 °C and 180 rpm in 50 mL conical centrifuge tubes. At an OD_600_ of 0.1, 135 μL of the exponentially growing cells was transferred to a 96-well microtiter plate. Whole-cell biosensor cultures were supplemented with 15 μL of cell-free supernatants of mandelate-producing strains. Subsequently, cells were incubated in the microplate reader with orbital shaking at 30 °C and 500 rpm. RFP fluorescence and optical density were measured every 5 min over the time course of 12 h using the same settings as for the single time point measurements.

### Production of Mandelate

Biosynthesis of mandelate was accomplished in *E. coli* DH5α Δ*tyrR* Δ*tyrA* carrying SBC009522, SBC009523, SBC009524, SBC009525, or SBC009527.^49^ Individual colonies of freshly transformed bacterial cells were used to inoculate 1 mL of TBP, supplemented with 0.4% glycerol and carbenicillin, in 96-deepwell blocks with breathable seals. After incubation overnight with orbital shaking at 850 rpm, 30 °C, and 75% humidity, seed cultures were normalized to OD_600_ = 1.0 before being diluted 1:50 into 1 mL of fresh TBP containing the respective supplements and returned to the shaker-incubator. At an OD_600_ of 1–1.5, cultures were induced by adding IPTG to a final concentration of 100 μM. Cultures were then returned to the shaker-incubator and samples were taken after 24 h. To obtain the cell-free supernatants, samples were clarified by centrifugation at 16,000 *g* for 10 min.

### Quantification of Target Compounds

A previously reported protocol was adopted to perform the quantification of S- and R-mandelate as well as phenylglyoxylate.^49^ Mandelate and phenylglyoxylate titers were first quantified using a rapid nonchiral method, followed by a chiral analysis of mandelate. The sample was prepared by diluting the cell-free supernatant 2-fold using 100% methanol. After a further 5-fold dilution using 10% methanol, the sample was thoroughly mixed by vortexing and transferred into a high-performance liquid chromatography vial using a 0.2 μm syringe filter.

UPLC analysis was performed using a 1290 Infinity II Agilent LC system equipped with a Waters ACQUITY BEH C18 column (50 mm × 2.1 mm, 1.7 μm) and a diode array detector measuring absorbance at 194 and 252 nm. The column was operated at 45 °C. The separation was achieved using a flow rate of 0.6 mL/min and a binary mobile phase consisting of A (H_2_O, 0.1% formic acid) and B (MeOH, 0.1% formic acid). The gradient elution program was 0–1.0 min, held at 80% A; 1.0–2.0 min, 80–5% A; 2.0–3.0 min, 5–80% A; 3.0–3.5 min, held at 80% A. All samples were kept at 10 °C throughout the analysis and the injection volume was 5 μL. Peak areas were integrated using Agilent OpenLab software. Metabolite concentrations were quantified using calibration curves generated from running standards of known concentrations which were prepared in the same manner as the samples. Chiral analysis was performed using a Waters ACQUITY UPLC H-Class System coupled to a Xevo TQ-S triple-quadrupole mass spectrometer (Waters), as previously reported.^49^

## Supporting Information Available

Further experimental details for plasmid construction; additional figures and tables as described in the text (PDF)
TFBMiner output data for selected compounds (XLSX)
