# Supplementary material for: TFBMiner: A User-Friendly Command Line Tool for the Rapid Mining of Transcription Factor-Based Biosensors
Source: ACS Synth Biol. 2023 Apr 13;12(5):1497–507. doi: 10.1021/acssynbio.2c00679 (PMC10204090; doi:10.1021/acssynbio.2c00679)
Supplement: Supplementary file 1 — sb2c00679_si_001.pdf [file sb2c00679_si_001.pdf]

Supporting Information

TFBMiner: A user-friendly command line tool for the rapid mining of transcription factor-based biosensors

Erik K. R. Hanko[1]*, Tariq A. Joosab Noor Mahomed[1], Ruth A. Stoney[1], and Rainer Breitling[1]

[1]Manchester Institute of Biotechnology, Faculty of Science and Engineering, University of Manchester, 131 Princess Street, Manchester M1 7DN, United Kingdom

*Author to whom correspondence should be addressed; Email: erik.hanko@manchester.ac.uk

Supplementary Methods

Plasmid construction

SBC015873 was constructed by Hifi DNA Assembly using NEBuilder Hifi. The synthesised gene fragment containing the codon-optimised *APZ15_29770* and the *APZ15_29770/APZ15_29775* intergenic region was combined with NdeI/AatII-digested pBbE8c-rfp.

SBC015875 was constructed by Hifi DNA Assembly using NEBuilder Hifi. The synthesised gene fragment containing the codon-optimised *C2L64_43720* and the *C2L64_43720/C2L64_43715* intergenic region was combined with NdeI/AatII-digested pBbE8c-rfp.

SBC015876 was constructed by Hifi DNA Assembly using NEBuilder Hifi. The synthesised gene fragment containing the codon-optimised *Pnap_1024* and the *Pnap_1024/ Pnap_1023* intergenic region was combined with NdeI/AatII-digested pBbE8c-rfp.

SBC015895 was constructed by Hifi DNA Assembly using NEBuilder Hifi. Oligonucleotide primers EH154_f and EH155_r were used to amplify the *APZ15_29770/APZ15_29775* intergenic region from the corresponding synthesised gene fragment. The PCR product was combined with NdeI/AatII-digested pBbE8c-rfp.

SBC015896 was constructed by Hifi DNA Assembly using NEBuilder Hifi. Oligonucleotide primers EH156_f and EH157_r were used to amplify the *C2L64_43720/C2L64_43715* intergenic region from the corresponding synthesised gene fragment. The PCR product was combined with NdeI/AatII-digested pBbE8c-rfp.

SBC015897 was constructed by Hifi DNA Assembly using NEBuilder Hifi. Oligonucleotide primers EH158_f and EH159_r were used to amplify the *Pnap_1024/ Pnap_1023* intergenic region from the corresponding synthesised gene fragment. The PCR product was combined with NdeI/AatII-digested pBbE8c-rfp.

SBC015877 was constructed by Hifi DNA Assembly using NEBuilder Hifi. Oligonucleotide primers EH160_f and EH161_r were used to amplify *APZ15_29770* from the corresponding synthesised gene fragment. The PCR product was combined with NdeI/BamHI-digested pBbB1k-rfp.

SBC015878 was constructed by Hifi DNA Assembly using NEBuilder Hifi. Oligonucleotide primers EH162_f and EH163_r were used to amplify *C2L64_43720* from the corresponding synthesised gene fragment. The PCR product was combined with NdeI/BamHI-digested pBbB1k-rfp.

SBC015934 was constructed by Hifi DNA Assembly using NEBuilder Hifi. Oligonucleotide primers EH164_f and EH165_r were used to amplify *Pnap_1024* from the corresponding synthesised gene fragment. The PCR product was combined with NdeI/BamHI-digested pBbA8k-rfp.

Supplementary Tables

Supplementary Table S1. Enzymatic chains returned for R-mandelate (KEGG COMPOUND ID C01983) with a chain length of ≤ 3 reactions.

Enzyme 1	Enzyme 2	Enzyme 3
EC 1.1.1.379	EC 4.1.1.7	
EC 1.1.1.379	EC 4.1.1.7	EC 1.2.1.28
EC 1.1.1.379	EC 4.1.1.7	EC 1.2.1.7
EC 1.1.1.379	EC 1.2.1.58	
EC 1.1.1.379	EC 1.2.1.58	EC 2.3.1.127
EC 1.1.1.379	EC 1.2.1.58	EC 2.3.1.220
EC 1.1.1.379	EC 1.2.1.58	EC 1.14.13.58
EC 1.1.1.379	EC 1.2.1.58	EC 1.3.7.8
EC 1.1.1.379	EC 1.2.1.58	EC 2.3.1.71
EC 1.1.1.379	EC 1.2.1.58	EC 2.3.1.144
EC 1.1.1.379	EC 1.2.1.58	EC 2.3.1.166
EC 1.1.1.379	EC 1.2.1.58	EC 2.3.1.196
EC 1.1.1.379	EC 1.2.1.58	EC 1.14.13.208
EC 1.1.1.379	EC 1.2.1.58	EC 2.3.1.177

Supplementary Table S2. Enzymatic chains returned for S-mandelate (KEGG COMPOUND ID C01984) for which output files were generated. Chain length of 3 enzymatic steps.

Enzyme 1	Enzyme 2	Enzyme 3	Number of operons with a score of 0
EC 1.1.99.31	EC 1.5.1.36	EC 1.1.99.31	14
EC 1.1.99.31	EC 1.5.1.36	EC 2.7.7.2	0
EC 1.1.99.31	EC 1.5.1.38	EC 1.1.99.31	20
EC 1.1.99.31	EC 1.5.1.38	EC 1.3.8.17	0
EC 1.1.99.31	EC 1.5.1.38	EC 2.7.7.2	0
EC 1.1.99.31	EC 1.5.1.38	EC 3.1.3.2	0
EC 1.1.99.31	EC 1.5.1.39	EC 1.1.99.31	0
EC 1.1.99.31	EC 1.5.1.41	EC 1.1.99.31	2
EC 1.1.99.31	EC 1.5.1.42	EC 1.1.99.31	0
EC 1.1.99.31	EC 1.13.12.16	EC 1.1.99.31	29
EC 1.1.99.31	EC 1.13.12.16	EC 1.2.1.3	0
EC 1.1.99.31	EC 1.13.11.79	EC 2.4.2.21	0
EC 1.1.99.31	EC 1.13.11.79	EC 2.5.1.54	0
EC 1.1.99.31	EC 1.13.12.16	EC 1.2.1.10	0
EC 1.1.99.31	EC 1.13.12.16	EC 2.7.7.2	0
EC 1.1.99.31	EC 1.13.12.16	EC 3.1.3.2	0
EC 1.1.99.31	EC 1.13.12.16	EC 3.1.3.102	0
EC 1.1.99.31	EC 1.13.12.16	EC 4.1.3.39	0
EC 1.1.99.31	EC 1.14.14.3	EC 1.1.99.31	0
EC 1.1.99.31	EC 1.14.14.5	EC 1.1.99.31	19
EC 1.1.99.31	EC 1.14.14.5	EC 1.2.1.3	0
EC 1.1.99.31	EC 1.14.14.5	EC 1.8.2.1	0
EC 1.1.99.31	EC 1.14.14.5	EC 1.8.4.10	0
EC 1.1.99.31	EC 1.14.14.5	EC 1.14.14.1	0
EC 1.1.99.31	EC 1.14.14.5	EC 2.7.7.2	0
EC 1.1.99.31	EC 1.14.14.5	EC 3.1.3.2	0
EC 1.1.99.31	EC 1.14.14.5	EC 3.1.3.102	0
EC 1.1.99.31	EC 1.14.14.181	EC 1.1.99.31	1
EC 1.1.99.31	EC 1.14.14.12	EC 1.1.99.31	3
EC 1.1.99.31	EC 1.14.14.12	EC 1.13.11.25	0
EC 1.1.99.31	EC 1.14.14.12	EC 2.7.7.2	0
EC 1.1.99.31	EC 1.14.14.12	EC 3.1.3.2	0
EC 1.1.99.31	EC 1.14.14.12	EC 3.1.3.102	0

EC 1.1.99.31	EC 1.14.14.34	EC 1.1.99.31	19
EC 1.1.99.31	EC 1.14.14.34	EC 1.8.2.1	0
EC 1.1.99.31	EC 1.14.14.34	EC 1.8.4.10	0
EC 1.1.99.31	EC 1.14.14.34	EC 1.14.14.1	0
EC 1.1.99.31	EC 1.14.14.34	EC 2.7.7.2	0
EC 1.1.99.31	EC 1.14.14.34	EC 3.1.3.2	0
EC 1.1.99.31	EC 1.14.14.34	EC 3.1.3.102	0
EC 1.1.99.31	EC 1.14.14.35	EC 1.1.99.31	0
EC 1.1.99.31	EC 1.14.14.155	EC 1.1.99.31	0
EC 1.1.99.31	EC 1.14.99.46	EC 1.1.99.31	2
EC 1.1.99.31	EC 1.14.99.46	EC 1.14.99.46	0
EC 1.1.99.31	EC 1.14.99.46	EC 2.7.7.2	0
EC 1.1.99.31	EC 1.14.99.46	EC 3.1.3.2	0
EC 1.1.99.31	EC 1.14.99.46	EC 3.5.1.110	0
EC 1.1.99.31	EC 4.1.1.7	EC 1.2.1.28	13

Supplementary Table S3. Putative biosensors for the detection of S-mandelate. Genes encoding the pathway for the conversion of S-mandelate into benzoate: gene 1 encodes (S)-mandelate dehydrogenase, gene 2 encodes phenylglyoxylate decarboxylase, and gene 3 encodes benzaldehyde dehydrogenase. Sensors with an ideal score of 0 were returned for 13 organisms.

KEGG Organism code	Organism	Locus tag gene 1	Locus tag gene 2	Locus tag gene 3	Locus tag regulator
BCEP	*Burkholderia cepacia* ATCC 25416 UCB 717	APZ15_29795	APZ15_29775	APZ15_29780	APZ15_29770
BSEM	*Burkholderia seminalis* FL-5-4-10-S1-D7	WJ12_29785	WJ12_29800	WJ12_29795	WJ12_29805
BURK	*Burkholderia* sp. JP2-270	DM992_18225	DM992_18245	DM992_18240	DM992_18250
BXE	*Paraburkholderia xenovorans* LB400	Bxe_B0136	Bxe_B0137	Bxe_C0302	Bxe_B0138
BXB	*Paraburkholderia xenovorans* LB400	DR64_5486	DR64_5487	DR64_8131	DR64_5488
BPH	*Paraburkholderia phymatum* STM815	Bphy_5156	Bphy_6157	Bphy_6158	Bphy_6156
PHS	*Paraburkholderia hospita* DSM 17164	C2L64_43710	C2L64_43715	C2L64_43695	C2L64_43720
PACS	*Paraburkholderia acidisoli* DHF22	FAZ98_17720	FAZ98_17725	FAZ98_17705	FAZ98_17730
KGY	*Kerstersia gyiorum* SWMUKG01	EHF36_00220	EHF36_00190	EHF36_13905	EHF36_00185
RHY	*Rhodoferax koreense* DCY110	RD110_17475	RD110_17450	RD110_17455	RD110_17445
PNA	*Polaromonas naphthalenivorans* CJ2	Pnap_1020	Pnap_1023	Pnap_1022	Pnap_1024
ACK	*Acidovorax* sp. KKS102	C380_23560	C380_23540	C380_23545	C380_23535
HRB	*Herbaspirillum rubrisubalbicans* M1	Hrubri_1083(mdlB)	Hrubri_1082	Hrubri_1085	Hrubri_1081

Supplementary Table S4. Bacterial strains used in this study.

Strain	Characteristic	Reference or source
E. coli 5α	K-12 *F− λ− fhuA2 Δ(argF-lacZ)U169 phoA glnV44 Φ80Δ(lacZ)M15 gyrA96 recA1 relA1 endA1 thi-1 hsdR17*	NEB (C2987-8)
E. coli 5α *ΔtyrR ΔtyrA*	5α *ΔtyrR ΔtyrA*	[1]
9522	*E. coli* DH5α *ΔtyrR ΔtyrA* carrying SBC009522	[1]
9523	*E. coli* DH5α *ΔtyrR ΔtyrA* carrying SBC009523	[1]
9524	*E. coli* DH5α *ΔtyrR ΔtyrA* carrying SBC009524	[1]
9525	*E. coli* DH5α *ΔtyrR ΔtyrA* carrying SBC009525	[1]
9527	*E. coli* DH5α *ΔtyrR ΔtyrA* carrying SBC009527	[1]

Supplementary Table S5. Oligonucleotide primers used in this study.

Primer name	Primer sequence (5' to 3')
EH154_f	tcattttcgccagatatcgacgtccgtgtcgaaccagattgaaagg
EH155_r	ttcgctactcgccatatggaat
EH156_f	tcattttcgccagatatcgacgtcgtcggaggggggaatatagac
EH157_r	ttcgctactcgccatatgca
EH158_f	tcattttcgccagatatcgacgtcttggcgaatattgaatagtccttgtag
EH159_r	ttcgctactcgccatatgttgaa
EH160_f	gatcttttaagaaggagatatacatatgtcctttaatctgcaacagttaatgatg
EH161_r	gatccttactcgagtttggatcctcaggataaggactgtccctct
EH162_f	gatcttttaagaaggagatatacatatgacaacggatttaaacttgattcgtac
EH163_r	gatccttactcgagtttggatcctcaaacgtcgctcaagcatt
EH164_f	gatcttttaagaaggagatatacatatgcatatcaatgacatcgacctg
EH165_r	gatccttactcgagtttggatcctcaagctaaatcccctgacttgt

Supplementary Table S6. Sequences of the synthesised DNA fragments. Gene coding sequences are shown in uppercase letters. AatII and NdeI restriction enzyme recognition sites are underlined.

Biosensor	Sequence
Burkholderia cepacia	tcattttcgccagatatc<u>gacgtc</u>TCAGGATAAGGACTGTCCCTCTTTGCGCAAA<br>CTTCCTGTAAGGTGACGAAGTTCGTCCAGCAGCTTAACTGCTGGA<br>GCGGGCAGAGTGCCACGACGGCGACGAAAGGCTGTTAAGGTACG<br>CTGTGCATCCAAACCTTCAATGTCCAGTGCGTCAATTACTCCGGCT<br>TTCGATTCCGTATCATACATGGGTTCGGCCATCCACGAGACGAAG<br>CCACAACGTGCTACCAAAGACTTCAGCACCGTCACTGAACGGGTT<br>TCTACAGCGATCGCCGGCATCGGAAGACCGCGGGCACGAAACGT<br>TTCTTGCATGTGCGCATAAGGTCCCGTGCCTTTAGGAGTTAAGGC<br>CCAGCGCTGGGTCAAAGTGTCGTCCAAGCGTAACCCGCGTTTCGC<br>ACGAAGCGGGTGGTCCAATGCAGCGACCACGAATGAACGGTCAG<br>TCCAACTGCAATCACTAATCGCAGCAACATCCTCGGTCTCGATCC<br>CTGAGGTACATAATGCAAGGTCAATTTCATAAGCAAGTAACCCTT<br>CGACCAAGCGATCCCAAACGCCTTCAATGACTTCTACACGCAAGT<br>TGGGCCACTTCTCAAGGACACAGGCGATAGCCATGGGCAGCACA<br>AGGGATGCGATGCTTCCGACCGCGCCAACCTTAATTACCCCCTTG<br>GCAAGCCCACGCATTGCGTCAAGCTCTTCGCGCGCCTGTTCGGCT<br>TCGCGTTGAAGGCTCATAGCGTGAGGCATAAGTGCTTCTCCAAGG<br>GCTGTCAGTTGCATACCTTTTGTGTGGCGCTCAAATAACGCACCA<br>CCGACAGACTCTTCCAAACGCTTGATCGAGCGCGACAGAGCTGGT<br>TGAGTCATGTTCAACATGGCAGCAGCGCGACCAAGCGAACCCGCT<br>GACACGATAGTAGTAAACATCATTAACTGTTGCAGATTAAAGGAC<br>ATcgtgtcgaaccagattgaaagggggaaaggggcgaacgagattccgggtcgcccggcgctagcta<br>ggtgtcttccctgcaagggctcgcggtgcccgctcattgcgaccaccgccgagcccccgacgacggcgaa<br>cagccttttccgacaaggcatccgggcggaattggccgatccggcattccgatagacgcatgccaaaatgta<br>atgactttgagctttttggcaattttcaaacagggtgccgaatcccatactgcggctcatcccatcaccgccag<br>cctgagtggcgaaccgcagaggattc<u>catatg</u>gcgagtagcgaa
Paraburkholderia hospita	tcattttcgccagatatc<u>gacgtc</u>TCAAACGTCGCTCAAGCATTGGGCAATCGTG<br>TCGCAGAACCACTGCTGGGCGGCAGAGTCCTCTGCATGAGGGTAC<br>CAATATAAATTCACCTCAAAGGGAGGAATTTCAAGGGGTAACGG<br>CAAAACGCGCATACGCCCGTCGCTCGCAAAACTGCGGGCGATGC<br>GCGTTGGAAGGGTCAGGACCAGGTCAGTTGTAGCAATGACAGCG<br>ACTAAGGAAGTAAAGTGCGGCACACGCAGAGAAATCTTACGAGT<br>CAACCCCATGCTCCCCATTACATCCTCTACTAAATGGTGGCCCGTA<br>AATGGAGCTACGACCACATGGTTGGCGGAAACGTAATCCGCAAG<br>GGTCATTGCGTTCCCAATGCTGGGATGTGTAGGTGACAGCAAACA<br>ACTATAAGATTCGGTGAACAGAGCTTTTTTGCGCACCTGGCCACC<br>CACAAATTGCAGATTCCCTACTACAGCGTCAACTGACCCTGTTTG<br>CAGCCATTCCGTAATACGCTCACTGTCGATTTGGACAATTTCCAAC<br>TCAACTGAAGGTGCAATTGCCTGCAACTCACGTACCAAGAAGGGC<br>AAGAAGTGCAATTCGCCAAGATCACTAAGAGCTAAGCGAAAGCA<br>ACGATTACTTCCAGTAGGATCGAACTGGCGCGTGGTGCTGATTGC<br>CGTCTCGATGGAATCCAAGGCGCTCTTAAATGCATTGAAAAGTTG<br>AGTAGCGTTGAAAGTAGGAACCATTCCGTCACGCGTGCGAGTGAA<br>CAATGGATCCTGAAGCAAGTCGCGCAGACGGTTCAGGGAATACG<br>ACACGGAGGGCTGGGTAATACTCAAACGACGCGCGGCTGCGCTC<br>ACTGAACGGGTCTCATAGATAGCGACGAAGGTACGAATCAAGTTT<br>AAATCCGTTGTCATgtcggagggggaatatagacacgttctattataagaacaaaaaaacgat<br>gattggatttatcgagcttcgctgcctagcatgagcctgttgatcaggaatattg<u>catatg</u>gcgagtagcgaa

Polaromonas naphthalenivorans	tcattttcgccagatatcgacgtcTCAAGCTAAATCCCCTGACTTGTCAGAAAAA<br>AGGGTTACGATTAACTGACGCAGCCATTGATTTCCTGGGTCCGCC<br>TCAAAACGACGCGACCAATGCAAACTTACAGTAAAGTCACGCAG<br>AGGGAACGGGGGCTCAATAATCGCGTAACCTCCTGCGGCGGCAA<br>AAAGTTTTGCAATGTTGCGAGGCATGACCACGGCCAGATCAGTCG<br>CCTTGACGATCGCCGGCAAAACCATAAAATGTTCAGTACCCAAAC<br>GCAGACGGTCCTCCAATTGAAGCAACTGCAGGATACGTAATGTGT<br>CACTATGAGTGCGCACTGCTACAAATTCAAGCTGGCGTAAGTCTC<br>CCAAAAGTGCCTGCCCCGAACGACGGCGACGTGTAAATGGATGG<br>CCGTCACGCAGAAGCACGATATAGCGATCTTTCAGCAGTTGGACA<br>CGCTGAGTGTCCTTAACAGTAGGCAGGAATCCGAAGGCGAAGTC<br>AATACGCCCCGAGTCTAACGCCTGCGCAATGTCGGAACTAGCTAA<br>GGGAAGCGTTTCGATGCGTACCCCTGGGGCAAGGCGACGTAAAG<br>CGACCATAAGGTCCGGCAAGAAACGAGCCTCCCCAATGTCTGACA<br>TGTGGATGCGAAAAACTTTCGCAGCCTCTGCAGGGTTGAACTGTG<br>CAGCTTCATTCAAAGCCTGCTCAACCGTTGCCAGGGCACTTTGGA<br>CTGCTAAGGCTAAACGTTCCGCACGCGGCGTAGGTGCGACGCCTC<br>CAGGCGCGCGGACAAATAAAGCGTCCTTTACTAACAGACGCAGG<br>CGTGTCAATCCCTGAGATGTTGCGGGCTGGGTTAAACCCAACAAC<br>TCCGCCGCGCGGCTTACTGAGCGCGCGCGGTAAACGGCGTCAAAC<br>ACACGTAATAAATTCAGGTCGATGTCATTGATATGCATttggcgaatattg<br>aatagtccttgtagtttattgatttatggaaccggctggccgaagatcaccgcctgaccgactggttctgcccca<br>cgccgccctgttactggagattcaacatatggcgagtagcgaa

Supplementary Table S7. Plasmids used and generated in this study.

Plasmid	Characteristic	Reference or source
pBbB1k-rfp	Kan^R, pBBR1, P_{trc} - *rfp*	[2]
pBbE8c-rfp	Cm^R, ColE1, P_{araBAD} - *rfp*	[2]
pBbA8k-rfp	Kan^R, p15A, P_{araBAD} - *rfp*	[2]
SBC009522	pBbB5a - PpMR - SyHMAS	[1]
SBC009523	pBbB5a - PpMR - TB1006-Ptrc - SyHMAS	[1]
SBC009524	pBbA5a - PpMR - SyHMAS	[1]
SBC009525	pBbB1a - PpMR - SyHMAS	[1]
SBC009527	pBbB1a - PpMR - TB1006-Ptrc - SyHMAS	[1]
SBC015873	pBbEc - P_{APZ15_29770} - *APZ15_29770** - P_{APZ15_29775} - *rfp*	This study
SBC015875	pBbEc - P_{C2L64_43720} - *C2L64_43720** - P_{C2L64_43715} - *rfp*	This study
SBC015876	pBbEc - P_{Pnap_1024} - *Pnap_1024** - P_{Pnap_1023} - *rfp*	This study
SBC015877	pBbB1k - *APZ15_29770**	This study
SBC015878	pBbB1k - *C2L64_43720**	This study
SBC015934	pBbA8k - *Pnap_1024**	This study
SBC015895	pBbEc - P_{APZ15_29775} - *rfp*	This study
SBC015896	pBbEc - P_{C2L64_43715} - *rfp*	This study
SBC015897	pBbEc - P_{Pnap_1023} - *rfp*	This study

*Optimised for *E. coli* codon usage.

Supplementary Figures

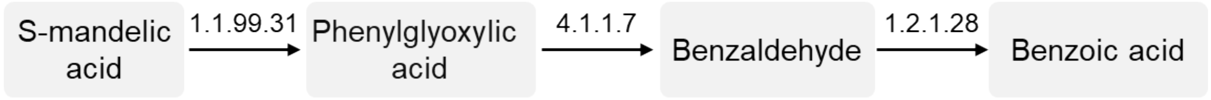

Supplementary Figure S1. Pathway for the conversion of S-mandelic acid into benzoic acid. Enzyme Commission numbers are indicated.

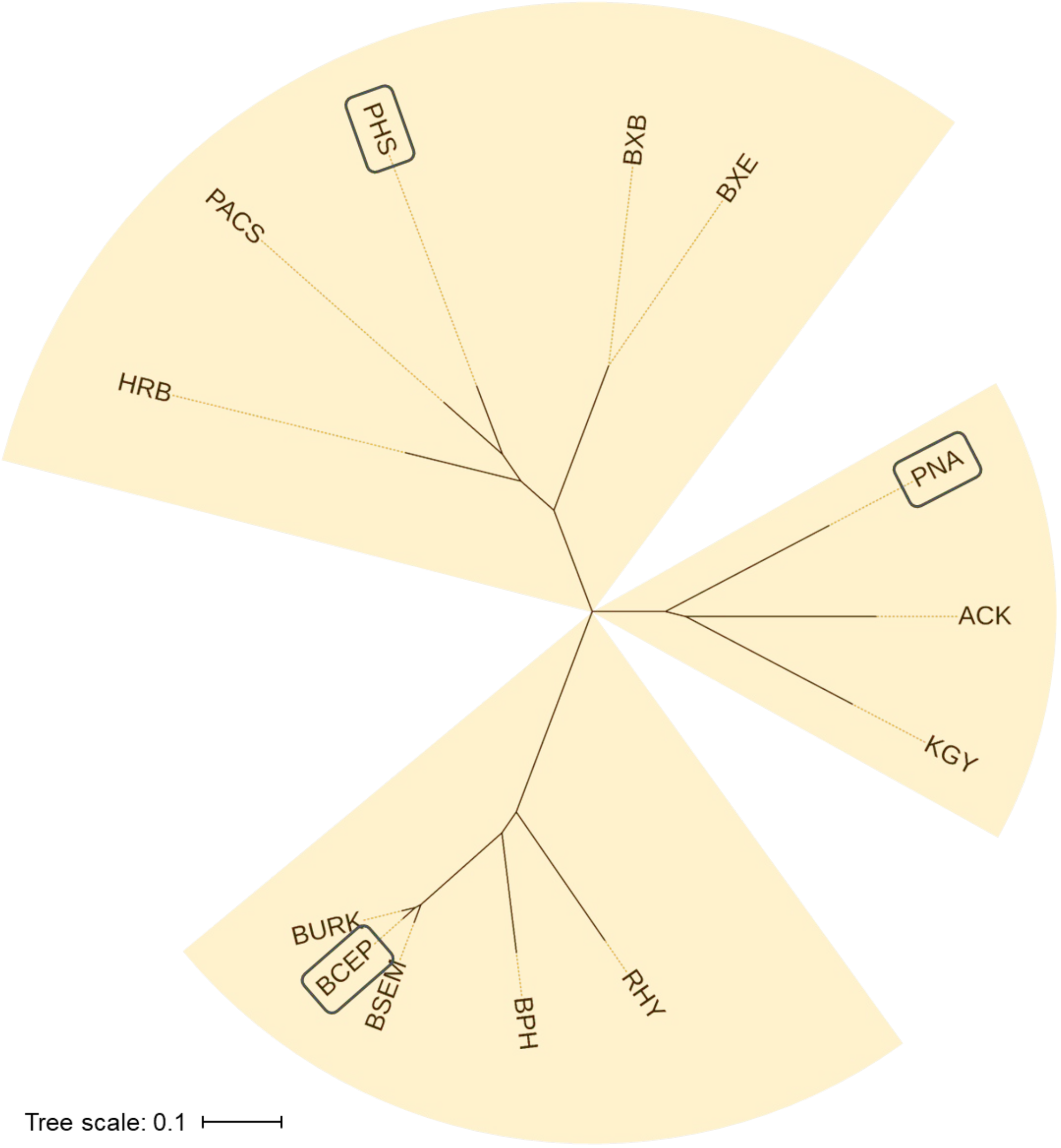

Supplementary Figure S2. Unrooted phylogenetic tree of putative mandelate-responsive transcriptional regulators derived from a protein sequence alignment of the 13 regulators listed in Table S3. Multiple protein sequence alignment was performed using ClustalW (URL: https://www.ebi.ac.uk/Tools/msa/clustalo/) and illustrated using iTOL[3]. Regulators that were experimentally tested for their ability to mediate gene expression in response to mandelate are highlighted.

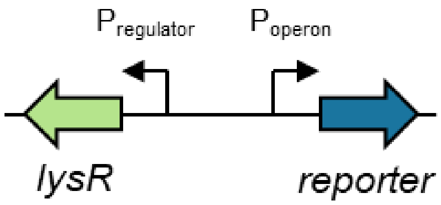

Supplementary Figure S3. Illustration of the biosensor. The intergenic region contains a putative bidirectional promoter mediating expression of regulator and reporter gene.

References

[1] Robinson, C. J., Carbonell, P., Jervis, A. J., Yan, C., Hollywood, K. A., Dunstan, M. S., Currin, A., Swainston, N., Spiess, R., and Taylor, S. (2020) Rapid prototyping of microbial production strains for the biomanufacture of potential materials monomers, *Metab. Eng. 60*, 168-182.

[2] Lee, T. S., Krupa, R. A., Zhang, F., Hajimorad, M., Holtz, W. J., Prasad, N., Lee, S. K., and Keasling, J. D. (2011) BglBrick vectors and datasheets: a synthetic biology platform for gene expression, *J. Biol. Eng. 5*, 1-14.

[3] Letunic, I., and Bork, P. (2021) Interactive Tree Of Life (iTOL) v5: an online tool for phylogenetic tree display and annotation, *Nucleic Acids Res. 49*, W293-W296.
